# Synthetic Food Preservatives Modulate Apoptotic Gene Expression in HepG2 Cells: Divergent Effects of Sodium Benzoate, Potassium Sorbate, and Sodium Metabisulfite

**DOI:** 10.3390/nu17213466

**Published:** 2025-11-03

**Authors:** Márton Pintér, John M. Macharia, Orsolya Liza Kövesdi, Nóra Rozmann, Afshin Zand, Katalin Szerb, Tímea Varjas, Bence László Raposa

**Affiliations:** 1 Doctoral School of Health Sciences, Faculty of Health Sciences, University of Pécs, Vörösmarty Mihály Str. 4, 7621 Pécs, Hungary; 2 Institute of Emergency Care, Pedagogy of Health and Nursing Sciences, University of Pécs, Vörösmarty Mihály Str. 4, 7621 Pécs, Hungary; 3 Department of Public Health Medicine, Medical School, University of Pécs, Vörösmarty Mihály Str. 4, 7621 Pécs, Hungary; 4 Faculty of Health Sciences, University of Pécs, Vörösmarty Mihály Str. 4, 7621 Pécs, Hungary; 5 Institute of Basics of Health Sciences, Midwifery and Health Visiting, Faculty of Health Sciences, University of Pécs, Vörösmarty Mihály Str. 4, 7621 Pécs, Hungary

**Keywords:** additive, preservatives, sodium benzoate, potassium sorbate, sodium metabisulfite, apoptosis, gene expression, epigenetics

## Abstract

**Background:** The accelerated lifestyle of modern society has increased reliance on processed foods preserved with synthetic additives. Although these substances effectively extend shelf life, several studies have raised concerns about potential adverse effects, suggesting that excessive or long-term exposure may interfere with essential cellular processes, including apoptosis. **Objectives:** This study aimed to investigate the impact of three widely used synthetic food preservatives; sodium benzoate (SB), potassium sorbate (PS), and sodium metabisulfite (SMB) on apoptosis-related gene expression in the human hepatocellular carcinoma cell line (HepG2). **Methods:** HepG2 cells were exposed to five increasing concentrations (6.25, 12.5, 25, 50, and 100 mg/L) of SB, PS, or SMB for 24 and 48 h. The transcriptional changes of key apoptotic genes (*CASP3*, *CASP8*, *BAX*, and *BCL2*) were quantified by real-time quantitative reverse transcription PCR (RT-qPCR) to evaluate their potential effects on intrinsic and extrinsic apoptotic pathways. **Results:** SB and PS induced dose-dependent transcriptional changes in apoptosis-related genes. Both preservatives upregulated *BAX* and downregulated *BCL2*, indicating an intrinsic pathway response, while simultaneously decreasing *CASP3* and *CASP8* expression associated with the extrinsic pathway. In contrast, SMB did not cause significant gene expression changes. **Conclusions:** SB and PS induced concentration- and time-dependent transcriptional alterations in apoptosis-related genes in HepG2 cells. In contrast, SMB did not elicit significant gene expression changes under the same conditions. These gene-level modulations were most evident at higher concentrations, which exceed typical dietary exposure levels. Therefore, while SB and PS were associated with transcriptional alterations at higher, experimentally relevant concentrations, additional research using primary human hepatocytes is needed to determine whether similar patterns occur in normal liver cells under physiological exposure conditions.

## 1. Introduction

The growing global dependence on processed and convenience foods reflects profound changes in dietary habits associated with industrialization and urban living. To maintain product stability and prevent microbial spoilage, synthetic preservatives have become indispensable components of the modern food supply. Nevertheless, increasing evidence suggests that frequent and long-term exposure to these additives may have subtle but biologically significant effects on cellular homeostasis, providing the rationale for the present study. However, most experimental studies have applied preservative concentrations far exceeding real-world dietary exposure levels, limiting their toxicological relevance. Therefore, contextualizing the tested doses with regulatory acceptable daily intake (ADI) values is essential for meaningful interpretation.

Currently, many food products contain synthetic preservatives such as SB, PS, and SMB to inhibit microbial spoilage and prolong shelf life [1,2]. These compounds are generally recognized as safe at permitted levels, which are regulated by food safety authorities such as the European Food Safety Authority (EFSA) and, at the international level, the Joint FAO/WHO Expert Committee on Food Additives (JECFA). Nevertheless, there is growing concern about adverse health effects that may arise from high-dose or long-term exposure [3,4,5]. The relationship between diet and cancer has drawn particular attention [6], as certain food additives may influence cellular processes linked to carcinogenesis for instance, by enhancing pro-inflammatory signaling pathways like nuclear factor kappa B (NF-κB) and tumor necrosis factor-alpha (TNF-α) [1,7]. In particular, some widely used preservatives have been reported to affect the mechanisms of programmed cell death (apoptosis) in mammalian cells [3]. Since evasion of apoptosis is a hallmark of cancer [8], any substance that perturbs apoptotic pathways could, in theory, contribute to tumor development or progression given sufficient exposure. Among these agents, SB has received particular scientific attention due to its extensive use and reported cellular effects, making it a relevant model compound for exploring how synthetic preservatives interact with apoptotic mechanisms.

Benzoic acid and its salts such as sodium, potassium, and calcium benzoates (E210–E213) are legally approved as food additives within the European Union and have undergone several toxicological assessments by international and European authorities. The Scientific Committee on Food (SCF) evaluated these compounds in 1994 and 2002, establishing a group acceptable daily intake (ADI) of 0–5 mg/kg body weight per day for benzoic acid and its salts, which also encompasses benzyl alcohol and related benzyl derivatives used as flavouring agents. These conclusions are in line with those of the Joint FAO/WHO Expert Committee on Food Additives (JECFA), which has assigned similar ADI values based on available toxicological data. The European Food Safety Authority (EFSA) currently maintains this ADI and continues to review benzoate exposure and safety in light of updated dietary and pharmacokinetic evidence. SB is used in acidic foods and beverages (e.g., sodas, fruit juices, condiments) for its antimicrobial properties. It is generally considered safe at typical dietary intake; for instance, an acceptable daily intake (ADI) of 5 mg per kg body weight per day has been established for benzoic acid and its salts. Although SB is generally considered effective and safe within permitted limits, several studies have also reported adverse biological effects under certain conditions. Notably, concentrations exceeding the ADI range (0–5 mg/kg body weight/day) have been shown to elicit oxidative stress and apoptotic signaling in vitro, suggesting that cellular effects may arise under supraphysiological exposure scenarios [9]. In vivo experiments in rodents have shown that repeated high-dose SB administration (in the range of hundreds of mg/kg/day) can induce oxidative stress and neurobehavioral deficit [10]. Genotoxicity assays have also found that SB causes DNA damage, evidenced by increased micronucleus formation and chromosomal breaks in cultured human lymphocytes [11] as well as in animal models [12]. Furthermore, in vitro studies suggest SB is not biologically inert—for example, Yilmaz and Karabay [3] reported that SB activated the NF-κB pathway and triggered apoptosis in cultured colon cancer cells in a dose-dependent manner. Likewise, another study found that chronic SB administration in mice led to pancreatic islet inflammation and β-cell apoptosis [13]. These findings raise concerns that excessive exposure to SB could disrupt cellular homeostasis and tilt the balance of cell survival and death pathways toward a pro-apoptotic state. Together, these data point to the possibility that other preservatives with similar chemical functions may exert comparable molecular effects, warranting a closer look at compounds such as PS.

PS is valued for its antifungal efficacy and remains safe within legal limits, yet recent work hints at subtler biological costs. Dietary sorbate shortened the lifespan of *Drosophila melanogaster* while increasing reactive oxygen species and caspase-linked cell loss in the insect gut [14]. In rats, sustained intake of PS elevated hepatic NF-κB activity and lipid peroxidation indices, suggestive of low-grade inflammation [1]. Data from human cells, however, are fragmentary and usually probe only one apoptotic branch. Whether sorbate can synchronously disturb both the mitochondrial and death-receptor apoptotic pathways—and at what doses—remains an open question; our study addresses this gap using human HepG2 liver cells. SMB is a sulfiting agent commonly added to foods like dried fruits, wines, and pickled products as an antioxidant to prevent browning and spoilage. Sulfite additives can cause allergic or asthmatic reactions in sensitive individuals [15], and they are known to generate oxidative stress at high concentrations [16]. Recent research suggests that SMB may also reduce cell viability and induce apoptosis in certain cell types [17]. On the other hand, some reports indicate minimal genotoxic effects of sulfites even at permitted concentrations, which correspond to an acceptable daily intake (ADI) of 0–0.7 mg SO_2_ equivalents per kg body weight per day, as established by JECFA and endorsed by the European Food Safety Authority (EFSA) [18]. Compared to benzoate and sorbate, the influence of SMB on cancer cell physiology—particularly with respect to apoptosis—remains underexplored. Since the liver is a primary site for detoxifying ingested sulfites, examining the effects of SMB on liver-derived cells could provide insight into whether this preservative poses a risk of altering apoptotic processes in hepatic tissue. For this reason, the human hepatocellular carcinoma cell line (HepG2) was selected as an in vitro hepatic model for this study. Although HepG2 retains several metabolic features of normal hepatocytes, it also exhibits altered apoptosis regulation and xenobiotic metabolism, which should be considered when interpreting the findings. This cell line is metabolically competent and widely applied in toxicological research because it retains several functional characteristics of normal hepatocytes, including phase I/II enzyme activity, protein synthesis, and active transport mechanisms. Therefore, it provides a relevant and well-established model for assessing how food additives such as SB, PS, and SMB may modulate cellular homeostasis and apoptosis-related gene expression.

Apoptosis is a tightly regulated form of programmed cell death that eliminates damaged or unnecessary cells without triggering inflammation. It proceeds via two major pathways: the intrinsic (mitochondrial) and the extrinsic (death-receptor) routes [19,20]. In the intrinsic pathway, cell fate depends on the balance between pro-apoptotic (e.g., *BAX*, *BAK*) and anti-apoptotic (e.g., *BCL-2*, *BCL-XL)* members of the BCL-2 family, which regulate mitochondrial permeabilization and the release of apoptogenic factors that activate downstream caspases [21,22]. A high *BAX*/*BCL*-*2* ratio is typically linked to intrinsic pathway activation. The extrinsic pathway, in contrast, begins when ligands such as FasL or TNF-α bind to their receptors, forming the death-inducing signaling complex (DISC) and activating caspase-8 (*CASP8*). *CASP8* subsequently triggers effector caspases—especially caspase-3 (*CASP3*)—initiating the execution phase of apoptosis and cleavage of essential cellular proteins [23]. Altered expression of key apoptotic genes (*BAX*, *BCL2*, *CASP8*, *CASP3*) thus reflects changes in a cell’s susceptibility to programmed death. Based on these mechanisms, this study examined how SB, PS, and SMB affect apoptosis-related gene expression in human HepG2 cells, a metabolically active hepatic model widely used in toxicological research. While previous studies have examined the cellular effects of individual food preservatives, comparative analyses assessing how multiple commonly used preservatives concurrently influence both intrinsic and extrinsic apoptotic regulators in a human hepatic model remain limited. Addressing this gap, the present study provides a molecular-level comparison that integrates dose- and time-dependent transcriptional responses relevant to food safety assessment.

## 2. Materials and Methods

### 2.1. Cell Culture

The experiments were conducted using the human hepatocellular carcinoma cell line HepG2 (ATCC HB-8065). Cells were cultured in high-glucose Dulbecco’s Modified Eagle’s Medium (DMEM; Thermo Fisher Scientific, Waltham, MA, USA) supplemented with 10% fetal bovine serum (FBS; Gibco, Thermo Fisher Scientific, USA) and 1% penicillin–streptomycin antibiotic mix (10,000 U/mL penicillin G, 10 mg/mL streptomycin; Sigma-Aldrich, St. Louis, MO, USA). Cultures were maintained at 37 °C in a humidified incubator with 5% CO_2_. Cells were routinely subcultured every 2–3 days to maintain exponential growth, by rinsing with phosphate-buffered saline (PBS; Merck, Darmstadt, Germany) and detaching with 0.05% trypsin–0.02% EDTA solution (Sigma-Aldrich). For experiments, cells were seeded into 6-well tissue culture plates (Corning Inc., Corning, NY, USA) at approximately 5 × 10^5^ cells per well and allowed to adhere for 24 h before treatment.

### 2.2. Preservative Treatments

Three common food preservatives were tested: SB (NaC_7_H_5_O_2_), PS (C_6_H_7_KO_2_), and SMB (Na_2_S_2_O_5_). All chemicals were of analytical grade purity (≥99%) and were obtained from Sigma-Aldrich. Stock solutions were freshly prepared by dissolving each preservative in sterile PBS, then further diluted into complete culture medium to achieve final concentrations of 6.25, 12.5, 25, 50, and 100 mg/L. This concentration range (6.25–100 mg/L) spans from values near estimated plasma levels following normal dietary intake to concentrations well above physiological exposure. The upper range was included to explore potential cytotoxic thresholds and mechanistic responses under stress conditions, consistent with previous in vitro toxicological studies [24]. The selected concentration range and exposure durations (24 and 48 h) were chosen based on previous scientific studies that employed similar experimental setups to evaluate the cytotoxic and apoptotic effects of food preservatives, allowing for reliable comparison and methodological consistency [25]. The present study focused exclusively on transcriptional responses, and no complementary assays (e.g., caspase activity, Western blotting) were performed. Therefore, the results reflect gene-level modulation rather than direct measurement of apoptosis execution.

Control cells received only the vehicle (culture medium without preservatives). All treatments and controls were set up in triplicate independent culture wells. Cells were exposed to the preservatives for either 24 h or 48 h. For the 48 h exposure groups, the treatment medium was replaced with fresh medium (containing the same preservative concentration) after 24 h to ensure continuous exposure. During treatment, cell morphology was monitored by inverted microscopy for any signs of cytotoxicity (e.g., cell rounding or detachment). At the end of the exposure period, the culture medium was gently removed, and cells were briefly rinsed with PBS to remove residual treatment. The PBS was immediately aspirated, and cells were lysed in situ for subsequent RNA extraction.

### 2.3. RNA Isolation and cDNA Synthesis

Total RNA was isolated from treated and control HepG2 cells using the ExtraZol Tri-reagent (Nucleotest Bio Kft., Budapest, Hungary) according to the manufacturer’s one-step acid guanidinium thiocyanate–phenol–chloroform extraction protocol [26]. In brief, cells in each well were directly lysed in 1 mL of Tri-reagent, and the lysate was homogenized and separated into phases by addition of chloroform (Sigma-Aldrich). The aqueous phase containing RNA was collected and precipitated with isopropanol (Merck, Darmstadt, Germany). The resulting RNA pellet was washed with 75% ethanol (prepared from absolute ethanol; BioTech Hungary, Budapest, Hungary), briefly air-dried, and dissolved in nuclease-free DEPC-treated water (Merck). RNA concentration and purity were assessed spectrophotometrically, confirming an A260/A280 ratio of approximately 2.0. The isolated RNA samples were stored at −80 °C until analysis. The thermal cycling profile included a reverse-transcription step at 42 °C for 5 min, followed by an initial denaturation at 95 °C for 3 min and 45 amplification cycles of 95 °C for 5 s, 56 °C for 15 s, and 72 °C for 5 s, with fluorescence acquisition at the end of each cycle. A final melt-curve analysis (95 °C for 5 s, cooling to 65 °C for 60 s, and a continuous ramp to 97 °C) was performed to verify amplicon specificity. Target genes (*CASP3*, *CASP8*, *BAX*, and *BCL2*) were quantified relative to the housekeeping gene HPRT1. Cycle-threshold (Ct) values for each gene were obtained in triplicate, and relative expression levels were calculated using the 2-ΔΔCt method, with untreated cells serving as the calibrator.

### 2.4. Quantitative PCR (qPCR)

Gene expression was quantified using a one-step reverse transcription quantitative PCR (RT-qPCR) approach, in which complementary DNA synthesis and amplification were performed within the same reaction mixture. Reactions were performed on a LightCycler 480 II real-time PCR system (Roche Diagnostics, Basel, Switzerland) equipped with a 96-well block. Each 20 µL reaction was prepared according to the kit instructions, including 5 µL of template total RNA and the provided one-step mastermix with reverse transcriptase. Gene-specific forward and reverse primer pairs (Integrated DNA Technologies, Coralville, IA, USA) were added to a final primer concentration of 0.2 µM; the primer sequences for the target genes are listed in Table 1. Thermal cycling 168 conditions consisted of an initial reverse transcription step at 42 °C for 5 min, followed by a 95 °C enzyme activation for 3 min. PCR amplification was then performed for 45 cycles of 95 °C for 5 s, 56 °C for 15 s, and 72 °C for 5 s. Fluorescence was measured at the end of each 72 °C extension phase. After cycling, a melting curve analysis was conducted (by heating from 65 °C to 97 °C) to verify the specificity of amplification products. The expression of apoptosis-related target genes (*CASP3*, CASP8, *BAX*, *BCL2*) was quantified relative to the housekeeping gene HPRT1. For each sample, threshold cycle (Ct) values were determined in triplicate. Relative mRNA expression changes were calculated using the 2ΔΔCT method [24], with the untreated control group as the calibrator.

### 2.5. Statistical Analysis

Data normality was verified using the Kolmogorov–Smirnov test. Since all gene expression (ΔCt) values followed a normal distribution, parametric tests were used for subsequent analyses. Two-way analysis of variance (ANOVA) was used to examine the effects of preservative concentration (0, 6.25, 12.5, 25, 50, and 100 mg/L) and exposure duration (24 h vs. 48 h) on the expression of each gene, including interaction terms between factors. When a significant main effect or interaction was found by ANOVA, group differences relative to control were further analyzed with Fisher’s least significant difference (LSD) post hoc tests, which are suitable for normally distributed parametric data and allow precise identification of treatment-specific differences without overly inflating Type I error rates. This approach provides greater statistical power and sensitivity compared to more conservative post hoc methods, making it appropriate for detecting subtle but biologically relevant gene expression changes. In cases where only two groups were compared (e.g., a specific dose at two time points), an independent-samples *t*-test was used. A significance criterion of *p* < 0.05 was applied for all statistical tests.

## 3. Results

### 3.1. Effects of Sodium Benzoate on Gene Expression

**Figure 1:**
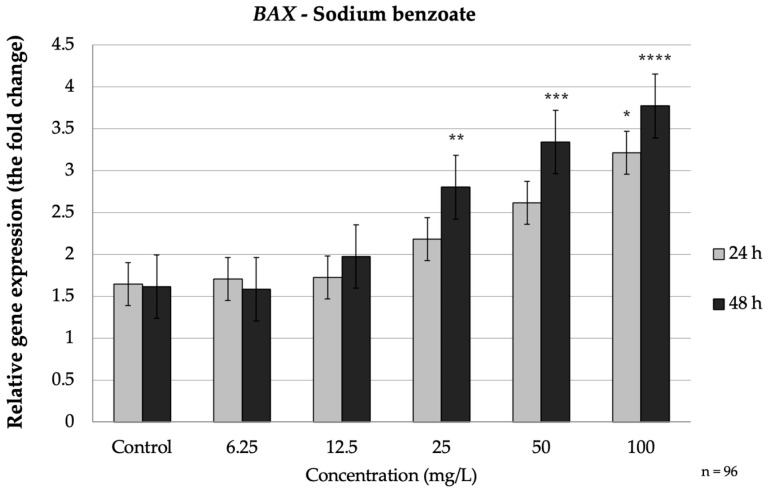
Relative mRNA expression levels of the apoptosis-related gene *BAX* in HepG2 cells following sodium benzoate exposure (6.25–100 mg/L) for 24 h and 48 h. Significant differences (*p* < 0.05) indicate expression changes compared with the corresponding control group at each time point. Data are presented as mean ± SD (*n* = 96). Statistical significance compared to control: * *p* = 0.018; ** *p* = 0.016; *** *p* = 0.001; **** *p* < 0.001.

SB did not markedly alter the expression of the apoptosis-related genes at 24 h, except at the highest concentration, but clear changes emerged after 48 h of exposure (Figure 1).

**Figure 2:**
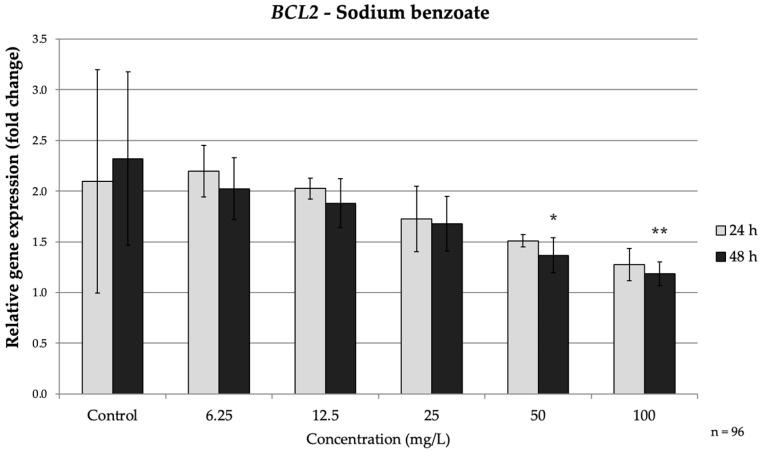
Relative mRNA expression levels of the apoptosis-related gene *BCL2* in HepG2 cells following sodium benzoate exposure (6.25–100 mg/L) for 24 h and 48 h. Significant differences (*p* < 0.05) indicate expression changes compared with the corresponding control group at each time point. Data are presented as mean ± SD (*n* = 96). Statistical significance compared to control: * *p* = 0.014; ** *p* = 0.005.

In the intrinsic pathway genes, *BAX* was up-regulated by SB in a time-dependent manner. After 24 h, only the 100 mg/L dose caused a significant increase in *BAX* mRNA (1.95-fold vs. control, *p* = 0.018). Upon prolonged exposure (48 h), *BAX* induction became pronounced at lower concentrations: SB at ≥25 mg/L significantly elevated *(p* = 0.016) *BAX* (peaking at 2.3-fold at 100 mg/L). Conversely, the anti-apoptotic *BCL2* showed a decreasing trend under SB treatment. No significant change in *BCL2* occurred at 24 h, but by 48 h the high-dose groups exhibited clear downregulation (Figure 2). At 50 mg/L and 100 mg/L SB, *BCL2* expression significantly decreased by approximately 1.7-fold (*p* = 0.014) and 2-fold, respectively (*p* = 0.005) (Figure 2). These reciprocal changes in *BAX* and *BCL2* indicate activation of the intrinsic (mitochondrial) apoptotic pathway over time with SB exposure, consistent with prior observations that preservative agents can shift the *BAX*/*BCL2* balance toward a pro-apoptotic state.

**Figure 3:**
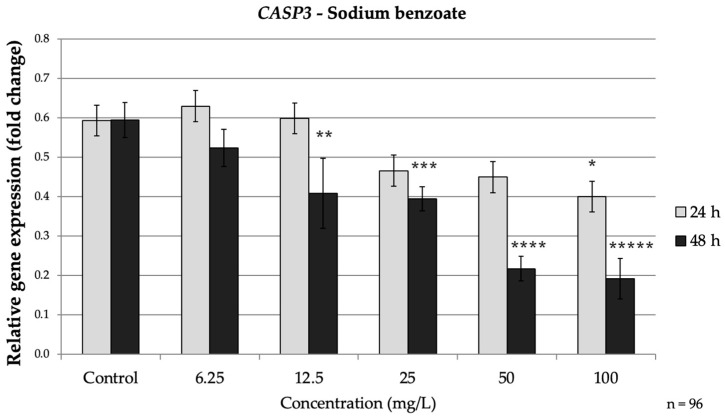
Relative mRNA expression levels of the apoptosis-related gene *CASP3* in HepG2 cells following sodium benzoate exposure (6.25–100 mg/L) for 24 h and 48 h. Significant differences (*p* < 0.05) indicate expression changes compared with the corresponding control group at each time point. Data are presented as mean ± SD (*n* = 96). Statistical significance compared to control: * *p* = 0.013; ** *p* = 0.028; *** *p* = 0.018; **** *p* = 0.001; ***** *p* < 0.001.

SB exposure also suppressed the expression of caspases associated with the extrinsic apoptosis pathway (caspases-3 and caspases-8), especially during the longer exposure time. At 24 h, caspases-3 showed minimal reduction (only a modest decrease to approximately 0.67-fold of the control at 100 mg/L, *p* = 0.013). In contrast, after 48 h *CASP3* was significantly down-regulated even at moderate SB doses, 12.5 mg/L SB reduced *CASP3* mRNA to approximately 0.69-fold of the control (*p* = 0.028.) Higher SB concentrations intensified this effect, with *CASP3* levels falling to approximately 0.37-fold of the control at 100 mg/L (*p* < 0.001) (Figure 3).

**Figure 4:**
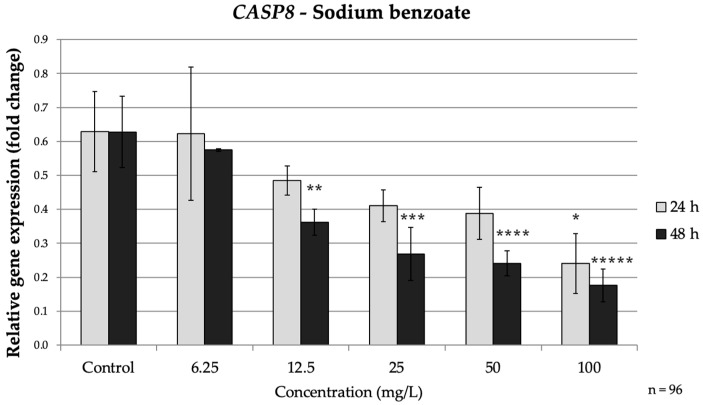
Relative mRNA expression levels of the apoptosis-related gene *CASP8* in HepG2 cells following sodium benzoate exposure (6.25–100 mg/L) for 24 h and 48 h. Significant differences (*p* < 0.05) indicate expression changes compared with the corresponding control group at each time point. Data are presented as mean ± SD (*n* = 96). Statistical significance compared to control: * *p* = 0.026; ** *p* = 0.006; *** *p* < 0.001; **** *p* < 0.001; ***** *p* < 0.001.

*CASP8* exhibited a similar pattern. By 48 h, *CASP8* expression was significantly lower than control at concentrations ≥ 12.5 mg/L; at 100 mg/L *CASP8* dropped to approximately 0.28-fold of the control (*p* < 0.001). At 24 h, only the highest SB dose had a notable impact on *CASP8* reducing it to about 0.28-fold of the control, (*p* = 0.026) (Figure 4). Taken together, prolonged SB exposure markedly down-regulated *CASP3* and *CASP8* while up-regulating *BAX* and down-regulating *BCL2*. This profile suggests that prolonged SB exposure promotes a shift from extrinsic toward intrinsic apoptotic signaling, consistent with a delayed, cumulative cellular stress response.

Overall, SB elicited predominantly time-dependent effects, with apoptosis-related genes showing stronger modulation after 48 h than at shorter exposure.

### 3.2. Effects of Potassium Sorbate on Gene Expression

**Figure 5:**
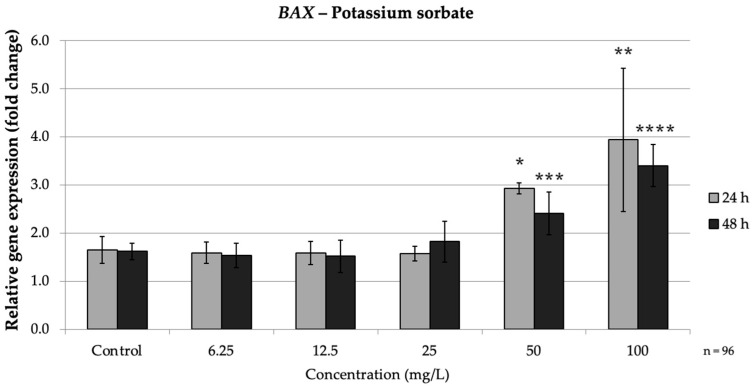
Relative mRNA expression levels of the apoptosis-related gene *BAX* in HepG2 cells following potassium sorbate exposure (6.25–100 mg/L) for 24 h and 48 h. Significant differences (*p* < 0.05) indicate expression changes compared with the corresponding control group at each time point. Data are presented as mean ± SD (*n* = 96). Statistical significance compared to control: * *p* = 0.008; ** *p* = 0.004; *** *p* = 0.003; **** *p* = 0.045.

PS exposure led to significant gene expression changes in a dose-dependent manner, evident even at 24 h for the highest concentrations and becoming more pronounced at 48 h (Figure 5).

**Figure 6:**
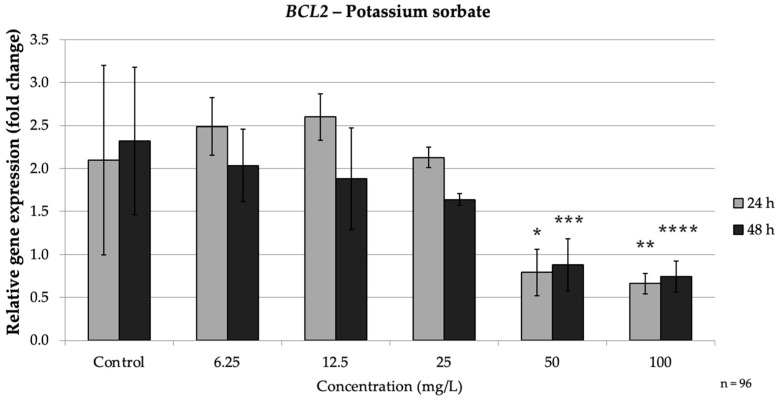
Relative mRNA expression levels of the apoptosis-related gene *BCL2* in HepG2 cells following potassium sorbate exposure (6.25–100 mg/L) for 24 h and 48 h. Significant differences (*p* < 0.05) indicate expression changes compared with the corresponding control group at each time point. Data are presented as mean ± SD (*n* = 96). Statistical significance compared to control: * *p* = 0.008; ** *p* = 0.004; *** *p* = 0.003; **** *p* = 0.045.

In the intrinsic pathway, PS significantly up-regulated *BAX* and down-regulated *BCL2*, especially at high doses. At both 24 h and 48 h, high PS concentrations (≥50 mg/L) markedly increased *BAX* expression while consistently suppressing *BCL2*, indicating activation of the intrinsic apoptotic pathway. Concurrently, *BCL2* was strongly repressed by PS treatment. At 24 h, 50 mg/L (*p* = 0.008) PS reduced *BCL2* expression to approximately 0.38-fold of the control, and 100 mg/L (*p* = 0.004) brought it down to approximately 0.32-fold of the control. Thus, high concentrations of PS simultaneously increased *BAX* and decreased *BCL2*, mirroring the pro-apoptotic intrinsic pathway pattern seen with SB exposure. These findings agree with reports that PS can trigger mitochondrial apoptotic signaling, as reflected by an increased *BAX*/*BCL2* ratio (Figure 6).

**Figure 7:**
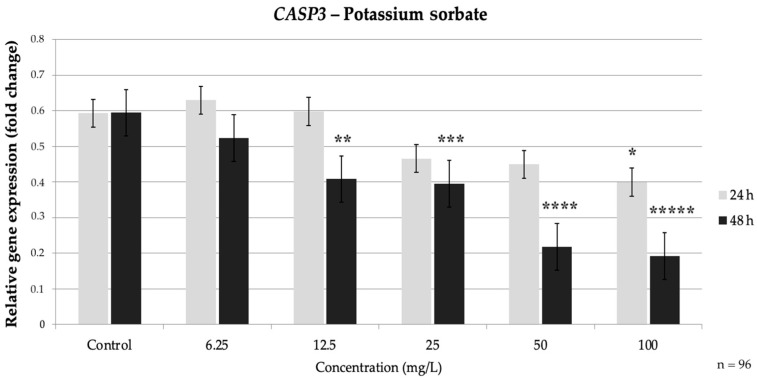
Relative mRNA expression levels of the apoptosis-related gene *CASP3* in HepG2 cells following potassium sorbate exposure (6.25–100 mg/L) for 24 h and 48 h. Significant differences (*p* < 0.05) indicate expression changes compared with the corresponding control group at each time point. Data are presented as mean ± SD (*n* = 96). Statistical significance compared to control: * *p* = 0.017; ** *p* = 0.004; *** *p* = 0.019; **** *p* = 0.006; ***** *p* < 0.001.

PS also produced a clear dose-dependent suppression of the extrinsic pathway caspases. At 24 h, *CASP3* showed a slight, non-significant dip at low doses; however, it dropped significantly at 50 mg/L (0.46-fold of the control) (*p* = 0.017) and even further at 100 mg/L to about 0.35-fold of the control, (*p* = 0.004). By 48 h, the *CASP3* reduction occurred at lower concentrations: 25 mg/L PS caused a significant 0.66-fold decrease (*p* = 0.019), and 50–100 mg/L drove *CASP3* down to roughly 0.57-fold and 0.37-fold of the control, respectively (*p* < 0.05) (Figure 7).

**Figure 8:**
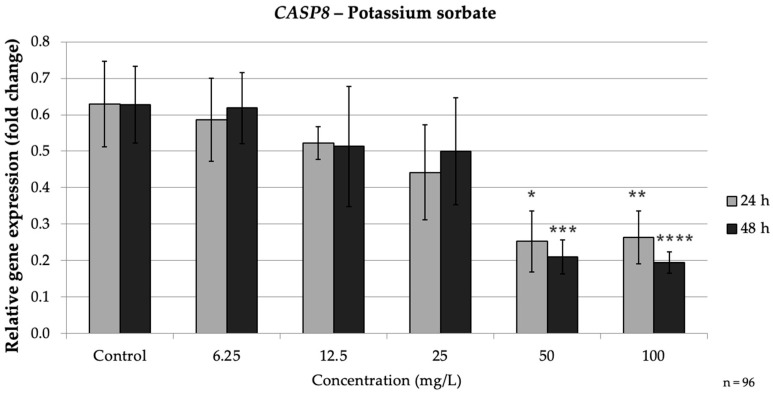
Relative mRNA expression levels of the apoptosis-related gene *CASP8* in HepG2 cells following potassium sorbate exposure (6.25–100 mg/L) for 24 h and 48 h. Significant differences (*p* < 0.05) indicate expression changes compared with the corresponding control group at each time point. Data are presented as mean ± SD (*n* = 96). Statistical significance compared to control: * *p* = 0.017; ** *p* = 0.02; *** *p* = 0.017; **** *p* = 0.013.

*CASP8* followed a similar trend. After 24 h, *CASP8* expression at 50 mg/L (*p* = 0.017) and 100 mg/L (*p* = 0.02) was less than 0.5-fold of the control. At 48 h, 50 mg/L (*p* = 0.017) PS reduced *CASP8* to approximately 0.33-fold, and 100 mg/L to about 0.31-fold of the control (*p* = 0.013), whereas lower doses (≤25 mg/L) did not significantly affect *CASP8*. These results demonstrate that PS, even over relatively short exposures, can significantly down-regulate caspase gene expression in a dose-dependent fashion, indicating a strong inhibitory effect on the extrinsic apoptotic pathway. Notably, the impact of PS was observed at earlier time points and was more tightly linked to dose compared to SB’s time-dependent effects, which suggests that PS may trigger a more immediate pro-oxidative and apoptotic response compared to SB. Taken together, PS induced a clear dose-dependent apoptotic response, becoming evident even at shorter exposures. This contrasts with the more time-dependent pattern observed for SB (Figure 8).

### 3.3. Effects of Sodium Metabisulfite on Gene Expression

In contrast to the above preservatives, SMB elicited no significant change in the mRNA expression of *BAX*, *BCL2*, *CASP3*, and *CASP8* in HepG2 cells at either 24 h or 48 h.

Two-way ANOVA analysis confirmed that none of the SMB-treated groups differed statistically from the time-matched controls for any of the four genes (*p* > 0.05). Minor, non-reproducible fluctuations were observed, without any consistent dose- or time-dependent pattern.

The absence of a marked response may reflect the rapid detoxification of sulfite ions by hepatic sulfite oxidase and the relatively high safety margin attributed to sulfite preservatives at short exposure durations.

Given the absence of meaningful changes, it appears that SMB had no measurable impact on the intrinsic or extrinsic apoptotic pathways in HepG2 under the conditions tested. This lack of effect likely reflects the high safety margin and efficient detoxification of sulfite preservatives in short-term cellular exposure, although other studies have noted that much higher doses or prolonged exposure to sulfites can induce cytotoxic and apoptotic effects in certain cell types [17].

Overall, the results indicate that while both SB and PS can modulate apoptosis-related genes in HepG2 cells, their modes of action differ SB’s influence is predominantly time-dependent (becoming evident after longer exposure), whereas PS provokes strong dose-dependent responses even at 24 h. By contrast, SMB up to 100 mg/L does not significantly alter the expression of these apoptotic markers in this model. These findings align with prior research showing that SB and PS may reduce cells’ susceptibility to apoptosis through down-regulation of extrinsic pathway mediators and simultaneous perturbation of intrinsic pathway regulators [1,2,27]. SMB, however, did not demonstrate any pro- or anti-apoptotic gene expression activity in HepG2 cells, highlighting a potential difference in the biological impact of this preservative compared to SB and PS. These results reinforce the notion that SB and PS, but not SMB, can modulate apoptotic gene expression in HepG2 cells through distinct time- and dose-dependent mechanisms.

### 3.4. Time-Dependent Changes in Gene Expression

The results of the independent-samples *t*-test revealed that significant time-dependent differences in relative gene expression were observed only for *CASP3* following exposure to SB among the tested preservatives. The mean expression levels at 24 h and 48 h differed significantly across several concentrations. For example, at 12.5 mg/L SB, the mean relative expression of *CASP3* after 24 h was significantly lower (*p* = 0.028) compared to 48 h. A similar pattern was observed at higher doses, where 24 h exposure resulted in a significantly greater reduction than at 48 h. These findings indicate that prolonged SB exposure consistently enhanced the downregulation of *CASP3* compared to shorter exposure (24 h), suggesting a stronger cumulative inhibitory effect on caspase-dependent apoptotic signaling over time. These findings suggest that the executioner caspase *CASP3* is particularly sensitive to cumulative SB exposure, supporting the time-dependent apoptotic pattern described earlier.

## 4. Discussion

Our data indicate that SB and PS modulate apoptosis-related gene expression in HepG2 cells through distinct yet converging transcriptional patterns. Both preservatives increased *BAX* expression and decreased *BCL2*, indicating activation of the intrinsic (mitochondrial) apoptotic pathway, consistent with earlier reports describing similar mitochondrial responses under preservative-induced oxidative stress [10,11,27]. In parallel, transcript levels of *CASP8* and *CASP3* were reduced, suggesting a partial suppression of the extrinsic apoptotic arm, a phenomenon also observed in other studies using benzoate derivatives [3,28]. In contrast, SMB did not significantly affect any of the examined apoptotic markers under the same experimental conditions. These results demonstrate that, at higher concentrations, SB and PS are not biologically inert but can induce coordinated transcriptional responses that influence the balance between pro- and anti-apoptotic signaling in hepatic cells.

The concurrent up-regulation of *BAX* and downregulation of *BCL2* observed in this study is consistent with oxidative stress-related mitochondrial responses previously reported for sodium benzoate and PS. Yilmaz and Karabay [3] demonstrated that SB exposure increased reactive oxygen species (ROS) formation and triggered apoptotic signaling in human colon cells, accompanied by elevated *BAX* and reduced *BCL2* expression. Similarly, Raposa et al. [2] and Abd-Elhakim et al. [1] observed mitochondrial oxidative stress and lipid peroxidation in hepatic tissues following preservative exposure, supporting a redox-mediated apoptotic mechanism. Several in vitro and in vivo studies have confirmed that exposure to SB or PS elevates ROS levels, induces lipid peroxidation, and impairs antioxidant defenses at higher concentrations [10,11,29,30,31,32]. Comparable increases in the *BAX*/*BCL2* ratio have been described in oxidatively stressed mammalian cells, indicating that mitochondrial dysfunction may represent a shared cellular outcome of excessive preservative exposure. In this context, the transcriptional shifts observed here likely reflect adaptive or stress-induced modulation of apoptotic signaling under redox-imbalanced conditions.

The concomitant downregulation of *CASP8* and *CASP3* observed in this study suggests that, despite upstream pro-apoptotic activation, downstream execution may be partially suppressed. This transcriptional pattern is consistent with previous findings showing that SB and PS can activate both apoptotic and pro-survival signaling cascades. Walczak-Nowicka and Herbet [5] highlighted the paradoxical role of benzoate, showing that it can act as a stressor activating inflammatory and anti-apoptotic pathways in neuronal systems. Moreover, Bandarian et al. [33] and Qu et al. [27] described that oxidative stress caused by benzoate or sorbate treatment can engage NF-κB-linked feedback mechanisms that attenuate caspase expression, resembling the transcriptional suppression we observed for *CASP3* and *CASP8*. In line with these reports, our results may reflect a compensatory survival response rather than a complete loss of apoptotic potential. Mersch-Sundermann et al. [4] also emphasized that HepG2 cells display distinct xenobiotic metabolism and stress adaptation compared with primary hepatocytes, which can explain the partial inhibition of caspase transcripts in this model. Additionally, Piper [28] and Abd-Elhakim et al. [1] proposed that preservative-induced oxidative imbalance can activate stress kinases and NF-κB simultaneously, resulting in dose-dependent modulation of apoptosis. Such biphasic behavior has been repeatedly observed under high-dose benzoate or sorbate exposure, where pro-apoptotic initiation is accompanied by incomplete execution due to NF-κB-driven anti-apoptotic feedback [5,20,34].

Emerging epigenetic evidence provides additional support for these findings. Transcriptome-wide analyses in HepG2 cells have shown that SB can reprogram the expression of numerous stress- and metabolism-related genes, exceeding the transcriptional impact of certain bio-preservatives such as nisin under comparable conditions [33]. Mechanistic studies indicate that preservatives can also modify chromatin structure: PS has been reported to induce histone lysine “sorbylation” through noncanonical class I HDAC activity, influencing inflammatory gene regulation [35], while benzoate exposure increases histone lysine benzoylation (K^bz^) and alters transcriptional output in mammalian cells [13]. In addition, post-translational protein modifications such as keratin-18 O-GlcNAcylation may modulate apoptotic signaling in epithelial systems [31]. These molecular alterations suggest that prolonged or high-dose preservative exposure can affect transcriptional programs controlling apoptosis and stress adaptation. This interpretation aligns with our observation that 48 h exposure intensified SB induced changes, PS produced strong, concentration-dependent transcriptional shifts. Collectively, these data imply that both preservatives may influence gene regulation not only through oxidative stress but also via chromatin-mediated mechanisms that shape the balance between pro- and anti-apoptotic responses [2,3,27,33,35,36].

The absence of significant effects of SMB on apoptotic gene expression in HepG2 cells is consistent with its relatively low cytotoxic potential under short-term exposure conditions. This observation agrees with reports showing that sulfite preservatives exert minimal effects in hepatic systems with active sulfite detoxification capacity. Hepatocytes, including HepG2 cells, express sulfite oxidase, which efficiently converts sulfite into sulfate, thereby limiting oxidative and apoptotic stress [18,19].

In contrast, studies conducted in cell types with low sulfite-oxidase activity—such as gastric or epithelial models have reported apoptosis, lipid peroxidation, and mitochondrial dysfunction following SMB exposure at higher concentrations or longer durations [14,15,16]. Therefore, the lack of measurable response in HepG2 likely reflects the high metabolic resilience of hepatic cells rather than a universal absence of SMB toxicity. Overall, these findings suggest that SMB’s biological impact is cell type and exposure dependent, in line with previous toxicological evaluations [17].

Genotoxicity data from previous studies support the interpretation that the transcriptional effects of SB and PS observed in our model occur primarily at high, supra-physiological concentrations. Several investigations in human lymphocytes have demonstrated DNA strand breaks, micronucleus formation, and chromosomal aberrations at concentrations in the tens to hundreds of µg/mL range, with SB generally showing greater potency than potassium benzoate, and PS inducing detectable genotoxicity only near its upper concentration limits [28,30,37,38]. Comparable findings from plant-based assays (e.g., Allium cepa) further confirm mitotic disturbances at elevated preservative exposures [39]. Consistent with these data, our results indicate that both SB and PS alter the expression of apoptosis-related genes only at higher concentrations, while lower doses remain largely inactive. These patterns suggest that the transcriptional changes detected here represent stress-related responses to excessive exposure, rather than effects expected within normal dietary intake levels. This conclusion aligns with toxicological evaluations reporting that both benzoates and sorbates are considered safe at their established acceptable daily intakes [9,34,36,40].

Evidence from animal and invertebrate studies further contextualizes the cellular effects of SB observed in our experiments. Chronic SB exposure in rats has been reported to impair reproductive function and induce oxidative stress, inflammation, and apoptosis in testicular tissue, accompanied by p53 activation and increased caspase-3 activity [41]. Other investigations have shown that even doses within the regulatory safety range can modulate redox and inflammatory signaling, including suppression of the Nrf2/HO-1 antioxidant pathway and activation of NF-κB-dependent inflammation; co-administration of zinc or antioxidants has been shown to attenuate these responses [42]. In pregnant animal models, high SB exposure has been associated with hepatic oxidative injury and mild genotoxicity, whereas in *C. elegans* models, low concentrations of SB shortened lifespan and increased lipid accumulation, possibly through interference with SKN-1/Nrf2-mediated redox regulation [12,43].

While interspecies differences limit direct extrapolation to humans, these findings collectively support the mechanistic interpretation derived from our HepG2 model: that SB can influence the balance between oxidative stress, inflammatory signaling, and apoptotic regulation under prolonged or excessive exposure. Importantly, these effects have not been observed at levels corresponding to typical human dietary intakes but emerge primarily under experimental conditions exceeding real-world exposure scenarios.

Beyond hepatic models, several studies have explored the potential neurobehavioral and immunological correlates of SB exposure. A randomized controlled community trial by McCann et al. [32] reported behavioral changes in children consuming a mixture of artificial colorants and SB, suggesting a possible interaction between dietary additives and neurobehavioral regulation, although the underlying mechanisms remain unclear and likely multifactorial [32]. From a biochemical perspective, SB has been shown to inhibit D-amino acid oxidase, leading to elevated D-serine levels and altered NMDA receptor signaling, as well as to promote Hippurate formation through glycine conjugation changes that may modulate neurotransmission at sustained exposures [5,28,44]. Experimental data also point to context-dependent immunomodulatory effects. In autoimmune encephalomyelitis models, SB administration mitigated T-cell-mediated inflammation and demyelination, likely via altered cytokine and leukocyte trafficking [45]. Conversely, chronic exposure to preservative mixtures in rodents produced shifts in cytokine balance (e.g., TNF-α, IFN-γ, IL-1β, IL-6, IL-10) without overt cytotoxicity [6]. These observations illustrate that SB can influence immune and neural signaling in a dose- and context-specific manner. Although such effects occur under experimental or disease-model conditions, they reinforce the broader pattern observed in HepG2 cells—namely, that benzoate exposure modulates redox and inflammatory pathways such as NF-κB. The convergence of these findings across systems highlights shared stress-response mechanisms rather than direct neurotoxic or immunotoxic outcomes at typical dietary intake levels.

Toxicokinetic data indicate that these preservatives are efficiently metabolized and eliminated in humans at typical dietary exposure levels. SB is rapidly conjugated with glycine to form hippuric acid, which is excreted in urine, while PS undergoes β-oxidation and related catabolic pathways. SMB is converted to sulfate by sulfite oxidase activity in the liver [34,36]. These detoxification processes maintain systemic concentrations well below those shown to alter apoptotic gene expression in vitro. The established acceptable daily intakes (ADIs)—5 mg/kg body weight for benzoates and 25 mg/kg for sorbates—provide a reference framework for interpreting the present findings [9,40]. Within these limits, human exposure levels are unlikely to reach the concentrations that produced measurable transcriptional changes in HepG2 cells. Nevertheless, our data highlight the value of integrating molecular endpoints, such as *BAX*/*BCL2* ratio and caspase expression, into future safety assessments to detect early, sub-cytotoxic alterations in stress and apoptosis pathways. Such endpoints can complement traditional toxicological measures and help clarify mechanistic thresholds relevant to chronic, low-dose exposure scenarios [33,35].

Overall, our findings demonstrate that SB and PS can modulate apoptosis-related gene expression in HepG2 cells in a concentration- and time-dependent manner, whereas SMB did not cause measurable transcriptional alterations under comparable conditions. These results should be interpreted with caution, as HepG2 cells represent a transformed hepatic phenotype with limited physiological relevance. Therefore, further studies using primary hepatocyte models and extended exposure durations are warranted to verify these molecular responses under more realistic biological conditions.

### Limitations

Some limitations of this study should be acknowledged. The experiments were performed in HepG2 cells, which—although cancer-derived—remain one of the most widely used in vitro hepatic models for mechanistic toxicology and nutritional gene expression research due to their stable phenotype, reproducibility, and metabolic competence. While only mRNA-level changes were analyzed in this study, this approach provides an established and sensitive method for detecting early transcriptional responses that precede measurable protein or phenotypic alterations. Several previous toxicogenomic studies have successfully applied similar RT-qPCR-based designs to characterize apoptotic and oxidative stress pathways in hepatic models without additional protein-level validation [2,3,27,33]. Nevertheless, integrating complementary assays—such as Western blotting, caspase activity analysis, or flow cytometric apoptosis detection—would further substantiate the mechanistic interpretation in future work. The applied concentrations intentionally extended beyond typical dietary exposure levels to identify potential molecular thresholds; therefore, the results should be interpreted within this experimental framework. Future studies using primary hepatocytes and chronic low-dose exposure models incorporating multi-endpoint validation are recommended to confirm and extend these findings under physiologically relevant conditions.

## 5. Conclusions

In conclusion, SB and PS were associated with dose- and time-dependent alterations in the expression of apoptosis-related genes (*BAX*, *BCL2*, *CASP8*, and *CASP3*) in HepG2 cells, while SMB showed no measurable changes up to 100 mg/L. The most pronounced transcriptional responses were detected after 24–48 h at 25–100 mg/L, concentrations exceeding typical dietary exposure levels. These findings offer molecular insight into how high preservative concentrations may transiently influence apoptotic and stress-related pathways in hepatic cells. Although the tested concentrations were above real-world exposure ranges, the observed transcriptional patterns emphasize the importance of evaluating chronic, low-dose effects in advanced hepatic culture systems to better clarify their physiological relevance.

## Figures and Tables

**Table 1:** Primer Sequences for RT-qPCR Amplification.

Gene Symbol	Full Gene Name	Forward Primer (5′ → 3′)	Reverse Primer (5′ → 3′)
*CASP3*	*Caspase-3*	CTG AGC CAT GGT GAA GAA G	CGG CAG GCC TGA ATA ATG
*CASP8*	*Caspase-8*	CCA GTG GGC AAG AGA ATT AG	CAA GTG ACC AAC TCA AGG G
*BAX*	*BCL2 associated X, apoptosis regulator*	GAG CTG CAG AGG ATG ATT G	GCC TTG AGC ACC AGT TT
*BCL2*	*BCL2 apoptosis regulator*	GGC CAG GGT CAG AGT TA	CCT CTC TTG CGG AGT ATT TG
*HPRT1*	*Hypoxanthine phosphoribosyltransferase 1 (HPRT1) (housekeeping control)*	TGC TTC TCC TCA GCT TCA	CTC AGG AGG AGG AAG CC

## Data Availability

The original contributions presented in the study are included in the article, further inquiries can be directed to the corresponding author.

## Abbreviations

The following abbreviations are used in this manuscript:

HepG2	human hepatocellular carcinoma
CASP3	caspase-3
CASP8	caspase-8
BAX	BCL2-associated X protein, Apoptosis Regulator
BCL2	BCL2 Apoptosis Regulator
NF-κB	nuclear Factor kappa-light-chain-enhancer of activated B cells
TNF-α	tumor Necrosis Factor-alpha
SB	sodium benzoate
PS	potassium sorbate
SMB	sodium metabisulfite
BAK	BCL2 Antagonist/Killer
BCL-XL	B-cell lymphoma—extra large
DNA	deoxyribonucleic acid
RNA	ribonucleic acid
ROS	reactive oxygen species
RT-qPCR	quantitative reverse transcription polymerase chain reaction
DMEM	Dulbecco’s modified eagle’s medium
FBS	fetal bovine serum
EDTA	ethylenediaminetetraacetic acid
RNase	ribonucleas
NMDA	N-methyl-D-aspartate receptor
DEPC	diethyl pyrocarbonate-treated
PCR	polymerase chain reaction
cDNA	complementary DNA
HPRT1	hypoxanthine phosphoribosyltransferase 1
Ct	Cycle-threshold
ΔCt	delta Cycle-threshold
mRNA	messenger ribonucleic acid
